# Mitochondria as central hubs in synaptic modulation

**DOI:** 10.1007/s00018-023-04814-8

**Published:** 2023-06-02

**Authors:** Filipe V. Duarte, Daniele Ciampi, Carlos B. Duarte

**Affiliations:** 1 CNC - Center for Neuroscience and Cell Biology, University of Coimbra, Coimbra, Portugal; 2 III - Institute for Interdisciplinary Research, University of Coimbra, Coimbra, Portugal; 3 IRCCS Humanitas Research Hospital, Rozzano, Milan, Italy; 4 Department of Life Sciences, University of Coimbra, Coimbra, Portugal

**Keywords:** Mitochondria, Synaptic regulation, Intracellular trafficking, Intracellular signalling, Cannabinoid receptors, Neurotrophins

## Abstract

Mitochondria are present in the pre- and post-synaptic regions, providing the energy required for the activity of these very specialized neuronal compartments. Biogenesis of synaptic mitochondria takes place in the cell body, and these organelles are then transported to the synapse by motor proteins that carry their cargo along microtubule tracks. The transport of mitochondria along neurites is a highly regulated process, being modulated by the pattern of neuronal activity and by extracellular cues that interact with surface receptors. These signals act by controlling the distribution of mitochondria and by regulating their activity. Therefore, mitochondria activity at the synapse allows the integration of different signals and the organelles are important players in the response to synaptic stimulation. Herein we review the available evidence regarding the regulation of mitochondrial dynamics by neuronal activity and by neuromodulators, and how these changes in the activity of mitochondria affect synaptic communication.

## Mitochondria in neurons

Even though the brain represents less than 2% of the body mass, it consumes approximately 20% of the total metabolic energy under resting conditions. Most of this energy is spent by neurons, mainly to reverse the alterations in ion gradients across the plasma membrane resulting from the generation of action potentials and synaptic transmission [1]. A single neuron contains hundreds to thousands of mitochondria [2], which function is indeed crucial to maintain neuronal integrity and function under normal physiological conditions. In particular, mitochondria are enriched at chemical synapses where neuronal communication takes place [3].

Since the glycolytic capacity of neurons is limited, they are highly dependent on aerobic oxidative phosphorylation through mitochondria for the production of energy [4]. Mitochondria are very efficient organelles in utilizing oxygen (O_2_) and substrates, mostly derived from glucose, to generate cellular energy in the form of adenosine triphosphate (ATP) [5]. The production of ATP by mitochondria is important for many events that take place at the synapse, such as for the activity of ion channels, pumps, receptors, Ca^2+^-induced fusion of synaptic vesicles with the presynaptic membrane to release neurotransmitters (exocytosis) and recycling of neurotransmitters. In particular, neuronal mitochondria play a key role in the maintenance of intracellular calcium homeostasis, either by direct calcium buffering or by providing ATP necessary for the activity of the plasma membrane Ca^2+^-ATPase, and indirectly by contributing with the energy required to maintain the Na^+^ gradients across the plasma membrane which is important for the activity of the Na^+^/Ca^2+^ exchanger [6–8]. In addition, the local synthesis of ATP by mitochondria is also important for exo- and endocytosis at the presynaptic terminal, as well as to refill synaptic vesicles and reuptake of neurotransmitters [9]. Therefore, it is not surprising that mitochondria are found pre-synaptically, as well as in the postsynaptic region, close to the sites where action potentials are generated by ion influxes in excitatory synapses [3]. Accordingly, it was estimated that more than half of the total mitochondria present in hippocampal neurons are located in the distal regions of the neurites [10]. Mitochondria are also involved in the regulation of lipid synthesis and ROS signalling, and mediate apoptotic cell death [11].

Mitochondria are formed by two membranes that may have contact sites. The outer membrane is permeable to ions and small molecules, and its permeability is highly regulated. The intermembrane space (IMS) contains a number of proteins critical for proper mitochondria and cell function which reach their location after crossing the outer mitochondrial membrane [12]. The inner mitochondrial membrane (IMM) displays a very low permeability, thus forming a tight barrier between the mitochondrial matrix and the neuronal cytoplasm. This membrane is equipped with a variety of ion channels and transporters, like the Ca^2+^ uniporter, K^+^-ATP channels, and Na^+^/Ca^2+^ exchanger, as well as mitochondrial enzyme systems like the electron transport chain (ETC) [13].

Mitochondria in neurons are thought to arise from the cell soma and usually form a dynamic network [14]. However, axonal mitochondria are much more dynamic than the mitochondria present in dendrites [15, 16]. In particular, axonal mitochondria are highly mobile in anterograde and retrograde directions [17], and this movement is mediated by kinesin and dynein motor proteins, respectively, that travel along microtubule tracks. Therefore, neuronal mitochondria can be placed and retained in neuronal segments with high metabolic demand, such as active growth cones and/or pre- and post-synaptic compartments [18]. Mitochondria clustered at the synapses constitute a discrete pool from their non-synaptic counterparts, exhibiting distinguishable morphological [19, 20], proteomic [21], and Ca^2+^ handling characteristics [22], and increased vulnerability to oxidative damage [23]. In both the mouse and human cortex, non-synaptic mitochondria are significantly larger than the organelles present within synaptic terminals, and non-synaptic populations demonstrated elongation when compared with synaptic mitochondria, which displayed a spherical morphology [20]. Moreover, it has been shown that mitochondrial proteins enriched at the synapse are involved in tricarboxylic acid metabolism, as well as in lactate and glutathione metabolism. In contrast, mitochondrial proteins associated with glucose, lipid, ketone metabolism, signal transduction, morphogenesis, protein synthesis and transcription were enriched in non-synaptic mitochondria [21]. These unique features are likely determined by the activity in the synaptic microenvironment.

Mitochondria have several copies of the mitochondrial genome that consists of a 16.5 kb circular DNA molecule [24] that, in turn, provides the template for 13 essential proteins of the respiratory chain. In a mitochondrion there are more than 900 proteins, most of them being encoded by the nuclear genome [24]. Several diseases are caused by inherited mutations in mitochondrial DNA (mtDNA) [25].

Despite the functional importance of mitochondria at the synapse, and the evidence showing a role for mitochondrial dysfunction in synaptopathies [9], the mechanisms involved in their regulation in the synaptic compartment, including mitochondrial metabolic function, signalling function, morphology, or location, are not completely understood. This review is focused on the regulation of synaptic mitochondria by synaptic surface receptors and the functional implications. We also point out important gaps in the knowledge in the field.

### The role of mitochondria at the synapse

As mentioned above, mitochondria serve many essential functions in the neuron, particularly at the synapse [26, 27]. At the presynaptic terminal, mitochondria are vital to the process of neurotransmitter release by supplying highly demanded ATP and by buffering local calcium content [28]. In addition, synaptic ATP depletion through mitochondrial inhibition has a dramatic effect on synaptic vesicle turnover and synaptic vesicle release. Indeed, alterations in the mitochondrial density and/or variations in the rate of mitochondrial oxidative phosphorylation have an impact on the ability to regenerate ATP pre-synaptically and, consequently, affect synaptic vesicles exocytosis [29].

Since the first electron micrograph of mitochondria [30], electron microscopy (EM) has been instrumental to understand the anatomy and ultrastructure of mitochondria at a nanoscale resolution. It is understood that the structural appearance of mitochondria is tightly linked to their functional state. This has been illustrated in studies comparing the ultrastructure of mitochondria in different types of axons, which showed that presynaptic mitochondria in axons from neurons with high-activity are larger and contain more densely packed lamellar cristae than those of low-activity neurons [31]. In a previous EM study, mitochondria in pre- and post-synaptic compartments of hippocampal neurons were compared and it was found that pre-synaptic mitochondria are usually smaller and darker than post-synaptic mitochondria, meaning the former are more electron dense and with higher activity reflecting higher demands of energy supply and Ca^2+^ buffering in the pre-synaptic compartment [32]. However, the 2D snapshot views of mitochondria did not allow to investigate their entirety in a spatial context, which is particularly relevant for understanding mitochondria in neurons that have elaborate morphologies, including long and convoluted axons and dendrites. Therefore, 3D studies have been developed in which the authors mapped mitochondrial network morphology and complexity in the mouse brain and confirmed that mitochondrial morphology differs between sub-cellular locations and that axonal and dendritic mitochondria are generally morphologically distinct [33].

The biogenesis of synaptic mitochondria takes place in the cell body of the neuron, and from this compartment mitochondria are transported along the axons or dendrites through the activity of specific motor proteins that travel along microtubule tracks. Interestingly, it was shown that synapse formation enhances the bi-directional transport of mitochondria [34]. Furthermore, it has been argued that synaptic mitochondria may be longer lived than mitochondria present in the soma of neurons or glial cells, presenting greater cumulative damage from oxidative stress. Accordingly, it was shown that synaptic mitochondria exhibited increased age-associated mtDNA mutations as well as decreased bioenergetic function compared with non-synaptic mitochondria [22, 35]. The specificity of synaptic bioenergetics and the synapse-related mitochondrial heterogeneity is not very well understood, but some considerations about the differences in synaptic mitochondria when compared to non-synaptic counterparts have been reviewed in [36]. A recent study, disregarding the synaptic versus non-synaptic difference though, has shown that mitochondrial long-lived proteins (LLPs) can persist for months in tissues harbouring long-lived cells, as in the case of brain and neurons [37]. The study has unveiled long-lived mitochondrial proteins as fundamental players in the regulation of mitochondrial architecture in neurons and in other long-lived cells (such as in the heart). Moreover, although not mentioning mitochondria specifically, another group reported that synapses with distinct protein lifetimes are differentially distributed in neurons and in brain regions [38]. However, it remains poorly understood why and how synaptic and non-synaptic mitochondria exhibit different properties. It has been suggested that the more limited ability of synaptic mitochondria to buffer the [Ca^2+^]_i_ may be due to the punctuate morphology, differently from the tubular mitochondrial morphology observed in other regions of neurons [15, 23]. Mitochondria grouped at the synapses constitute a discrete pool from their non-synaptic counterparts, exhibiting distinct morphological, proteomic, enzymatic, and Ca^2+^ handling properties [9]. These distinctive features are likely determined by the activity in the synaptic compartment. Synaptic activity results in high Ca^2+^ influxes and demands instant ATP supply; the synaptic mitochondrial pool may thus have adapted its ability to buffer Ca^2+^ to better support synaptic functions.

Among neuronal mitochondria, the population present at the synapse appears to be the most susceptible to regulation by extracellular signals. In particular, the localisation of mitochondria at presynaptic sites can be altered during long-term activity changes, by a mechanism dependent on the Ca^2+^-sensing function of the mitochondrial trafficking protein, Miro1 [39]. Miro1-mediated activity-dependent synaptic repositioning of mitochondria allows neurons to homeostatically alter the strength of presynaptic Ca^2+^ signals in response to prolonged changes in neuronal activity. The latter results support a model in which mitochondria are recruited to presynaptic terminals during periods of raised neuronal activity and are involved in rescaling synaptic signals during homeostatic plasticity. The effect of neuronal activity on the distribution of mitochondria has an impact on neurotransmitter release since presynaptic Ca^2+^ signals are significantly decreased in terminals containing mitochondria, with a consequent downregulation of exocytosis [18, 40].

Presynaptic loss of dynamin-related protein 1 (Drp1), a central mediator of mitochondrial fission (see below), also impairs synaptic vesicle release [41], further supporting a role for the presynaptic organelle in exocytosis. Accordingly, the ATP supply from presynaptic localized mitochondria is important to fuel the assembly of the actin cytoskeleton [42]. In addition, the ATP content also affects the clustering of synaptic vesicles and mitochondria at the synapse [43], as well as the efficient mobilization of synaptic vesicles into the readily releasable pool [44]. Furthermore, mitochondria offer an additional dimension for the control of exocytosis through Ca^2+^ uptake from the cytosol. Neuronal activity causes large intracellular Ca^2+^ rises in the presynaptic compartment, mainly to act locally and to facilitate exocytosis of synaptic vesicles or to influence many signalling cascades. Mitochondria, thanks to the negative membrane potential created via the electron transport chain (ETC) along with plasma membrane Ca^2+^-ATPases and the ER, modulate these processes by regulating [Ca^2+^]_i_ levels [45]. Still, not all presynapses contain mitochondria, and their importance in maintaining neuronal homeostasis has been increasingly recognized [46]. In fact, the maintenance of a pool of presynaptic mitochondria was shown to increase the stability of presynaptic strength by maintaining the local ATP homeostasis [47].

The exposure of synaptic mitochondria to extensive [Ca^2+^]_i_ fluctuations may increase the risk for oxidative stress and Ca^2+^ accumulative damage [48]. Additionally, upon inhibition of the ETC complex I, synaptic mitochondria exhibit a more drastic reduction in respiration rates and ATP production when compared with non-synaptic mitochondria [49]. These results indicate that a synapse-specific tailoring of the mitochondrial population is needed for regular neuronal function. It also points to an important role in the maintenance of a proper mitochondrial proteome among different brain regions and neuron types (excitatory versus inhibitory neurons). Moreover, a functional cross-talk between energy sensing and mitochondria anchoring, in particular at the pre-synapse level, has been recently reported [18]. Synaptic activity induces AMP-activated protein kinase (AMPK) activation within the axon followed by the stimulation of the p21-activated kinase (PAK) to recruit mitochondria; this AMPK-PAK signalling pathway triggers the phosphorylation of myosin VI, which promotes mitochondrial recruitment and anchoring on the presynaptic terminals, thus maintaining presynaptic energy supply and calcium clearance during intensive synaptic activity.

Postsynaptic mitochondria are also important for local functions, as experimentally disrupting dendritic mitochondria leads to the loss of dendritic spines and their synapses [50]. Accordingly, it is acknowledged that mitochondria malfunction and consequent synaptic dysfunction contribute to a wide range of neurological defects [51].

## Mitochondrial morphology and dynamics

Mitochondria are highly dynamic cellular organelles, with the ability to change size, shape, and position over the course of a few seconds. Many of these changes are related to the ability of mitochondria to undergo the highly coordinated processes of fission (division of a single organelle into two or more independent structures) or fusion (the opposing process). These actions occur simultaneously and continuously in response to different stimuli, and the balance between them regulates the overall morphology of mitochondria within any given cell. It is well recognized now that mitochondrial elongation may be elicited either by excessive fusion or defective fission, and mitochondrial fragmentation may be driven by excessive fission or defective fusion [52]. Both fission and fusion are active processes that require many specialized proteins, including mechanical enzymes that physically alter mitochondrial membranes, and adaptor proteins that regulate the interaction of these mechanical proteins with organelles [53]. Although not fully understood, alterations in mitochondrial morphology appear to be involved in several activities that are crucial to the health of cells [54]. Indeed, the functional versatility of mitochondria is intimately related to a continuous reshaping of the cellular mitochondrial network in a series of processes, collectively referred to as mitochondrial dynamics and involving organelle fusion and fission as well as ultrastructural remodelling of the membrane [53].

Mitochondria display distinct morphologies and distributions in the axon and in dendrites. For example, in pyramidal neurons, the main excitatory neuronal subtype in the cerebral cortex, dendritic mitochondria display long and tubular shapes, forming a complex network filling 70–80% of the dendritic arbour. Mitochondria occupy a large portion of the dendritic arbour and are frequently close to spines [55]. In contrast, axonal mitochondria display a remarkably standard size and are small and punctate, occupying < 10% of axonal volume. In neurons, just as in any given cell type, mitochondrial morphology is controlled mostly by fusion and fission mechanisms, collectively termed mitochondrial dynamics [54]. Therefore, one would hypothesize that the striking differences in mitochondrial morphology between the axon and dendrites are the result of a high level of fission in the axon and a high degree of fusion in dendrites. Indeed the fission-dependent regulation of presynaptic mitochondrial size, as well as of axon branching, has already been shown in cortical pyramidal neurons [56].

Mitochondrial fission and fusion processes are both mediated by large guanosine triphosphatases (GTPases) belonging to the dynamin family, which are well conserved between yeast, flies, and mammals. Their combined actions divide and fuse the two lipid bilayers that surround mitochondria [57]. The mitochondrial inner membrane, which encloses the matrix, is folded into cristae that contain membrane-bound oxidative phosphorylation enzyme complexes and the bulk of the soluble electron transport proteins, whereas the smooth mitochondrial outer membrane (OMM) encapsulates the inner membrane and an intermembrane space [53]. Fusion allows mitochondria to compensate for one another’s defects by sharing components and thereby helps maintaining energy output in the face of stress [58]. There is evidence that mitochondrial dynamics, and especially mitochondrial fission, is regulated by calcium [59, 60]. It has been shown that IMM constriction is calcium-dependent and occurs at mitochondria-ER contact sites [61]. This mechanism is further inhibited by the loss of the mitochondrial calcium uniporter (MCU), also leading to mitochondrial elongation [62].

Fission segregates the most seriously damaged mitochondria to preserve the health of the mitochondrial network, in addition to regulating morphology and facilitating mitochondrial trafficking. The highly dynamic mitochondrial fusion and fission cycle is proposed to balance two competing processes: compensation of damage by fusion and elimination of damage by fission. Failure of these stress responses may lead to neuron death and neurodegenerative disorders [63].

Mitochondrial morphology and function (i.e., morpho-function) are interlinked in mammalian cells (extensively reviewed in [64]), and changes in mitochondrial morphology impact neuronal mitochondrial Ca^2+^ uptake and synaptic transmission [56], positioning mitochondria as neuromodulators.

### Mitochondrial fusion

In humans, there are two large nuclear-encoded dynamin-like GTPases, Mitofusin 1 (Mfn1) and Mitofusin 2 (Mfn2), that have both their N-terminus and C-terminus exposed to the cytosol. Mitofusins harbour a GTPase domain close to the N-terminus that is involved in the GTP hydrolysis, is required for their oligomerization (homo- and heterotypic oligomers) and promotes fusion of the outer membranes of adjacent mitochondria [65]. Mfn2 also includes a proline-rich domain involved in protein–protein interactions [66].

Fusion of the IMM occurs almost simultaneously with fusion of the OMM and is mediated by the GTPase Optic atrophy 1 (OPA1) [67]. OPA1 is anchored to the IMM by its N-terminal, and upon fusion of the OMM, OPA1 is cleaved into two forms: a long (L) form and a short (S) form. This proteolytic cleavage is carried out by metalloproteases YME1L and OMA1 in the mitochondria IMS [68]. Cleavage of OPA1 generates numerous protein isoforms consisting of higher molecular weight membrane-anchored L-forms (L-OPA1) and non-membrane- anchored S-forms (S-OPA1) [69].

Propper equilibrium of mitochondrial dynamics is particularly important in neurons as mutations in fusion and fission proteins cause several neuropathies and impaired development of the nervous system. As such, alterations in mitochondrial dynamics have been observed in many neurodegenerative disorders [70]. The importance of mitochondrial fusion proteins in neurons is highlighted by the fact that mutations in Mfn2 are often responsible for autosomal dominant Charcot-Marie-Tooth (CMT) disease [71, 72], which is a common peripheral neuropathy, while mutations in OPA1 are often the cause of autosomal dominant optic atrophy (ADOA) [73]. Moreover, several major neurodegenerative diseases, including Parkinson's, Alzheimer's and Huntington's disease, involve disruption of mitochondrial dynamics [74]. In the early stage of AD, studies have revealed that there is a greater increase in Drp1 compared to Marf (homologous to human Mfn2) expression. However, with disease progression, both Mfn2 and Mfn1 levels are markedly downregulated in the brain of AD patients [75, 76]. Furthermore, it has been shown that synapses and neuronal plasticity are also affected by mutations in OPA1 and Mfn2 with a consequent dysregulation of mitochondrial fusion [77–79].

### Mitochondrial fission

Mitochondrial fission involves the division of a single mitochondrion into two separate daughter mitochondria. Fission is mediated by a cytosolic dynamin family member: Drp1 in worms, flies, and mammals, and dynamin 1 (Dnm1) in yeast. The GTP-hydrolyzing enzyme Drp1 is recruited from the cytosol to form spirals around mitochondria that constrict to split both inner and outer membranes. The resulting constriction of mitochondrial tubules facilitates fission. Mitochondrial fission 1 protein (Fis1), mitochondrial fission factor (Mff), mitochondrial dynamics protein 49 and 51 (MiD49 and MiD51) are proteins of the mitochondrial outer membrane that act as Drp1 receptors to recruit the fission protein, often at sites where mitochondria contact with the endoplasmic reticulum (ER) [80]. Mitochondrial fission and fusion machineries are regulated by proteolysis and posttranslational modifications, and also by the formation of contact sites with other organelles, which provide the ability to integrate signals coming from several pathways. Some of the best-known posttranslational modifications affecting mitochondrial fission are the phosphorylation of Drp1 in different serine residues [53]. Mitochondrial fission is essential for growing and dividing cells to populate them with adequate numbers of mitochondria [63].

**Fig. 1:**
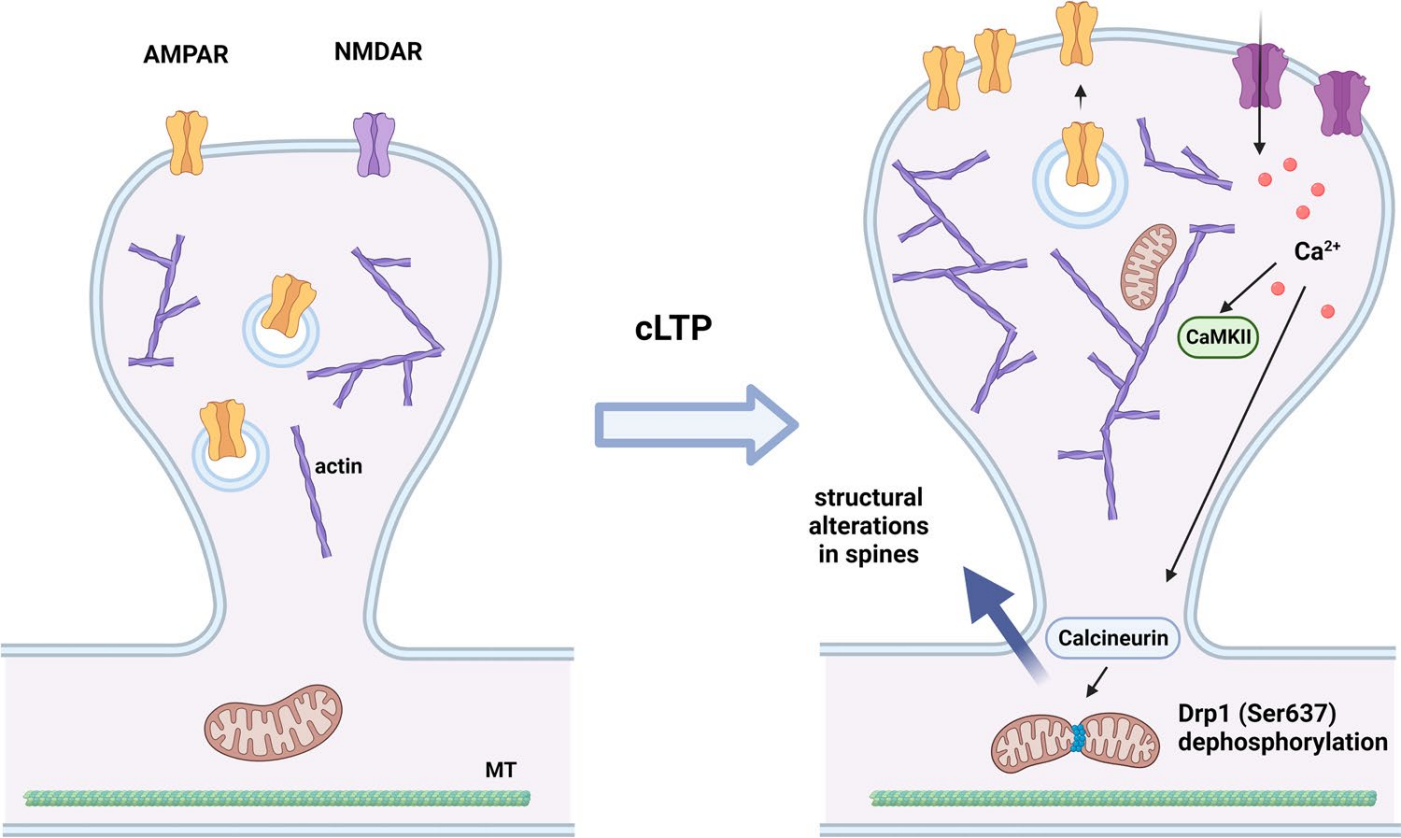
Alterations in mitochondrial dynamics upon induction of long-term synaptic potentiation (LTP). Chemical LTP (cLTP) is dependent on the activation of NMDA receptors coupled to an increase in the density of microfilaments and enhancement of spine volume, as well as the translocation of AMPA receptors to the synapse, with a consequent upregulation in their surface expression. These events rely on a rapid upregulation in dendritic mitochondrial fission, accompanied by an increase in mitochondrial matrix Ca^2+^ content. The increase in the fission of dendritic mitochondria is triggered by the increase in the [Ca^2+^]_i_, and mediated by the activation of Ca.^2+^- and calmodulin-dependent protein kinase II (CaMKII). (Created with BioRender.com)

Initiation of mitochondrial fission involves contact between the ER and mitochondria-mediated by actin cytoskeletal proteins such as spire-type actin nucleation factor 1 (Spire1c) and inverted formin 2 (INF2) [81], which carry out constriction of the mitochondria. Mitochondrial fission is modulated by post-translational modifications of Drp1, one of the most studied pro-fission proteins, that is phosphorylated in different possible sites coupled to differential functional responses [53]. As an example, mitochondrial fission is activated when Drp1 is dephosphorylated by the phosphatase calcineurin on Ser637, a process that renders Drp1 more active and that is regulated by the [Ca^2+^]_i_ [82] (briefly depicted in Fig. 1), as well as by phosphorylation of Drp1 on Ser616. Upon recruitment, Drp1 forms oligomeric rings, which promote mitochondrial scission by GTPase hydrolysis-mediated conformational changes that constrict the rings. Although the mechanisms surrounding OMM fission are well documented, the mechanisms driving IMM fission are less understood. The IMM protein mitochondrial fission process 1 (MTFP1) appears to mediate IMM fission. Overexpression of MTFP1 increases mitochondrial fission, while its loss leads to mitochondrial fusion [83]. Moreover, MTFP1 is a modulator of Drp1 phosphorylation [84]. Other proposed mechanisms of IMM fission are reviewed in detail in [60].

The functions of mitochondria are regulated by mitochondrial Ca^2+^ and by their dynamics, including fission, fusion and motility [85]. Fission of mitochondria is important in neuronal development and its dysregulation has been implicated in several neurological and psychiatric disorders [70]. Drp1 knockdown affects mitochondrial function and dendritic growth, compromises synapse formation and maintenance, and impairs synaptic transmission and memory formation [54]. Furthermore, cultured neurons lacking Drp1 could not maintain normal levels of mitochondria-derived ATP when energy consumption was enhanced by neuronal activity [86]. A recent study also showed that the induction phase of NMDA (N-methyl-D-aspartate) receptor-dependent long-term synaptic potentiation (LTP) triggers a rapid burst of dendritic mitochondrial fission coupled to the induction of structural changes in spine morphology [87]. Furthermore, it was observed that an increase in Drp1 in dendrites of D1 medium spiny neurons is associated with the reduced mitochondrial length and both cellular and behavioural plasticity during early abstinence after repeated cocaine administration [88].

### Mitochondrial trafficking

The importance of proper mitochondrial trafficking in neurons arises from their exceptional highly polarized cellular morphology. Whereas most cells are measured in micrometres or tens of micrometres, neurons extend their axons and dendrites for millimetres, centimetres, and, in the case of human peripheral nerves or corticospinal tracts, up to a meter. Thus, the neuron poses an extreme case for mitochondrial distribution and the need to supply energy to distant cellular regions. This challenge probably accounts for the fact that mutations in certain motor proteins ubiquitously expressed selectively affect neurons, thereby causing neurological pathologies in humans [89, 90]. The stress on the axonal transport system is such that perturbations that are innocuous to other cell types cause degeneration in neurons.

Long-distance transport of mitochondria along axons and dendrites is mediated by motor proteins that travel along microtubule tracks [5, 15, 42, 91, 92]. Studies performed in cultured neurons showed that the orientation of microtubules along the dendritic compartment is rather heterogeneous. Thus, in the proximal region of dendrites, closer to the soma, about half of the microtubules are oriented with their plus end out, while about 90% of the microtubules show this orientation in the distal region of dendrites [93]. This orientation contrasts with the organization of microtubules in the axonal compartment, with the plus end oriented towards the distal region of the axon [93–95]. The difference between the organization of microtubules in the dendritic and axonal compartments leads to a major difference in the contribution of motor proteins to the anterograde transport of mitochondria in the two compartments. Given the mixed orientation of microtubules along dendrites, kinesin and dynein could both play a role in the anterograde transport of mitochondria in this compartment. However, the available evidence suggests that dynein is the major motor protein involved in the transport of mitochondria toward the distal tip of the dendrites [96] (reviewed in [15]). In contrast, given the polarity of microtubules in axons, the anterograde transport of mitochondria in this compartment is mediated by kinesin, while dynein drives the retrograde transport [92] (reviewed in [15]).

**Fig. 2:**
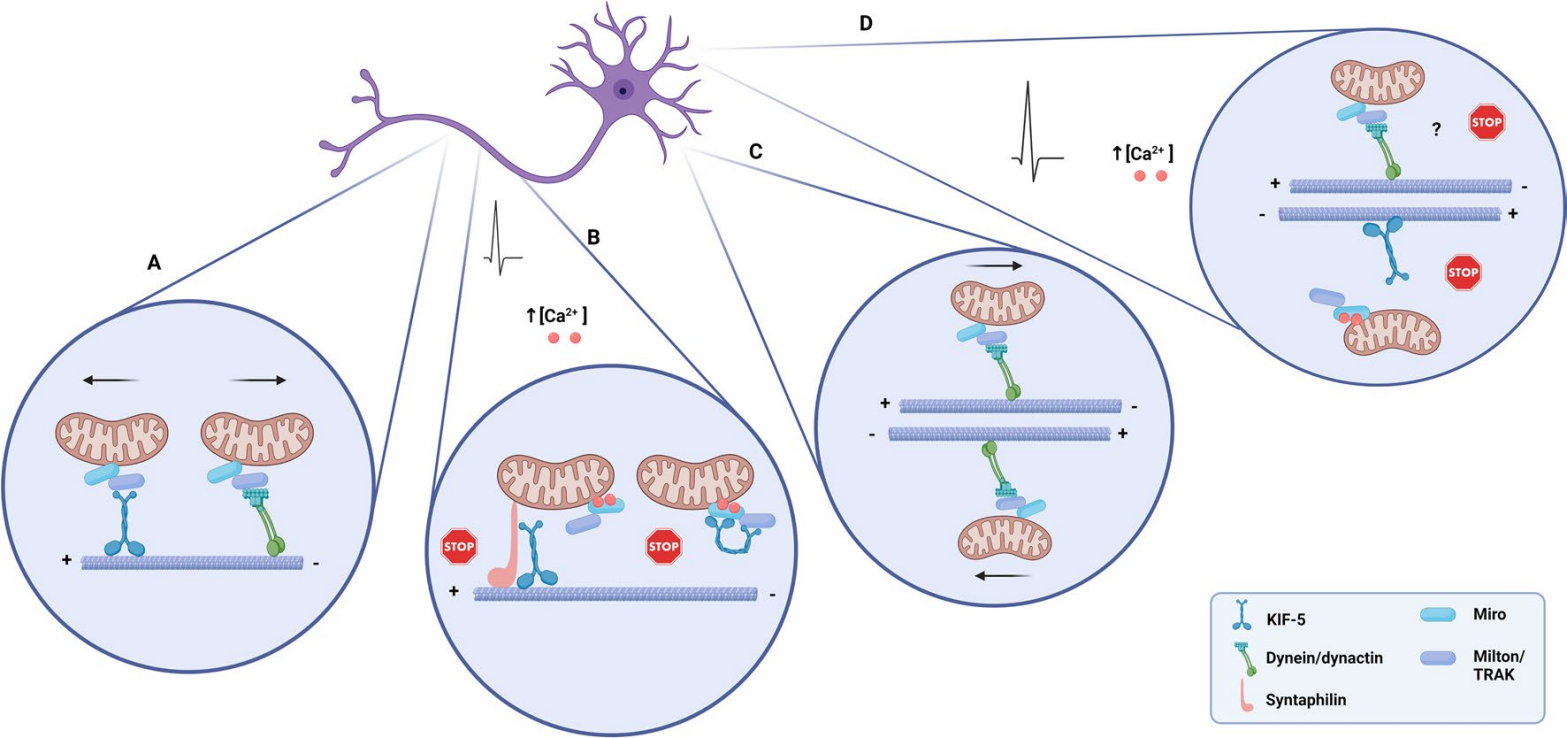
Mitochondrial trafficking in neurons. **A** Microtubules in the axon are uniformly organized with the plus-ends arranged away from the soma. This results in kinesins driving anterograde transport and dyneins mediating retrograde transport in axons. Trafficking and docking behaviour of mitochondria are mediated by the MOM proteins Miro and Milton (TRAK). **B** Activity-dependent increase in calcium modulates the trafficking adaptor proteins Milton and Miro, resulting in the disconnection of the motor protein kinesin either from the microtubules or the mitochondria. The neuron-specific mitochondrial protein syntaphilin anchors mitochondria to microtubules. Syntaphilin-based anchoring could also alter motor adaptor complex components in response to neuronal activity. **C** The dendritic microtubules exhibit a mixed arrangement, being dynein the primary motor protein in dendritic mitochondrial transport. **D** In the dendrite, an activity-dependent increase in calcium elicits a similar modulation of the adaptors Miro-Milton (TRAK) and consequent detachment of mitochondria from the microtubules; regarding the dynein-operated mitochondrial trafficking in dendrites, the role of calcium in arresting mitochondria is yet elusive. (Created with BioRender.com)

The trafficking of mitochondria along microtubules within the dendritic compartment, both in the anterograde and retrograde modes, depends on the activity of the dynein motor protein [96] together with the Miro-1 (mitochondrial Rho GTPase)-Milton/TRAK1/2 complex [91, 97, 98]. TRAK1 can bind both kinesin and the dynein/dynactin complex, while TRAK2 favours association with dynein/dynactin. In general, TRAK1 is broadly axonally localised and TRAK2 primarily mediates dendritic trafficking [96]. Accordingly, studies performed in hippocampal neurons isolated from Miro1^−/−^ mice showed reduced mobility of dendritic mitochondria [99], and similar results were obtained upon TRAK2 knockdown [92, 96–100]. Miro-1 is a protein of the mitochondrial outer membrane that binds to TRAK2 in dendrites, and the latter protein interacts preferentially with dynein via the p150^Glued^ subunit of dynactin. However, how this complex drives selectively the transport in the anterograde and retrograde modes along dendrites remains to be determined. TRAK2 also binds kinesin and this interaction may account for the effect of anti-kinesin antibodies, which inhibit the transport of mitochondria in the dendritic compartment [101]. Many signalling pathways converge on the Miro/TRAK complex, which allows mitochondrial positioning to be tailored to the local environment [101–106]. The neuron-specific mitochondrial protein syntaphilin anchors mitochondria to microtubules. Syntaphilin-based anchoring could also alter motor adaptor complex components in response to neuronal activity [47, 107] (depicted in Fig. 2).

Local transport of organelles in neurons, including mitochondria, is mediated by myosin motors that carry specific cargo along actin microfilaments. This is also the case for the transport of mitochondria in axon terminals and dendritic spines. Accordingly, actin microfilaments were shown to play an important role in the presynaptic accumulation of mitochondria in cultured chick sympathetic neurons during intense and repeated synaptic firing [108]. Studies performed in cultured cerebrocortical neurons showed that Myosin 6 is a motor protein that promotes the accumulation of mitochondria in presynaptic boutons in response to synaptic activity [18] (see also section The role of mitochondria at the synapse). Similarly, depolarization of the membrane induces the translocation of mitochondria into dendritic spines of cultured hippocampal neurons by an actin-dependent mechanism [109].

## Regulation of dendritic mitochondrial dynamics at glutamatergic synapses

Mitochondria are not detected in most dendritic spines within the cerebral cortex and hippocampus in the adult brain [33, 111], and a similar distribution is observed in cultured hippocampal neurons [109]. However, there are exceptions, such as the dendritic spines of CA3 pyramidal neurons [112] and olfactory bulb granule cells [113], which frequently contain mitochondria. Depolarization of cultured hippocampal neurons with KCl was found to increase the ratio of fission versus fusion and to enhance the mitochondrial distribution in dendritic spines [109]. The activity-dependent recruitment of mitochondria into spines is controlled by Wiskott-Aldrich syndrome protein (WASP)-family verprolin homologous protein 1 (WAVE1), a key regulator of actin polymerization that acts through activation of the Arp2/3 complex [110].

Different studies also showed that the transport of mitochondria along dendrites is regulated by neuronal activity. Thus, during the development of the primary visual cortex the motility of mitochondria is correlated with the pattern of neuronal activity. In particular, during the first two postnatal weeks mitochondria motility within the dendritic compartment decreases with the increase in the frequency of neuronal activity [114]. The transport of mitochondria along dendrites of cultured cerebrocortical and hippocampal neurons is arrested following stimulation of NMDA receptors, which are coupled to the influx of Ca^2+^ [101, 115]. This Ca^2+^-induced immobilization of mitochondria near the synapse may provide locally the energy necessary to cope with the increased demand resulting from the activity of glutamatergic synapses. Accordingly, spines depleted of mitochondria show an impairment in plasticity mechanisms and in translation activity [28, 109].

The Miro molecule has two Ca^2+^-binding EF-hands [116], making it a good candidate to play a role in the Ca^2+^-mediated effects in the transport of mitochondria. In the dendritic compartment, these effects can be initiated by NMDA receptor activation at the synapse, while in axons it can result from the voltage-dependent entry of Ca^2+^ following depolarization of the plasma membrane [101, 115, 117]. Indeed, neuronal expression of Miro mutants lacking the EF hands blocked the Ca^2+^-dependent arresting of mitochondria in dendrites [101] and axons [106]. However, the mechanism whereby Ca^2+^ stops the dynein-mediated transport of mitochondria in the dendritic compartment remains to be investigated. The available evidence concerns the effects of Ca^2+^ on the transport of mitochondria-mediated by kinesins, which is more relevant in the axonal compartment [92]. In this context, two models were proposed: (i) Ca^2+^-binding to Miro induces a conformational change in the protein which favours the interaction with the N-terminus of kinesin, thereby preventing the interaction of the motor protein with the microtubule track [106]; (ii) Ca^2+^ may disrupt a direct interaction between Miro and kinesin, thus detaching kinesin from mitochondria [101]. The former model was proposed based on studies performed on the regulation of the axonal transport of mitochondria, while the latter hypothesis was raised based on studies performed in dendrites, suggesting that distinct mechanisms of regulation may be in place in the two compartments. A recent study performed in *C. elegans* identified metaxins 1 and 2 (MTX1 and MTX2) as core components of the complexes of adaptor proteins involved in the transport of mitochondria [118]. Particularly, a role was identified for the MTX-2/Miro-1/TRAK2 complex in dynein-mediated mitochondrial trafficking. Knockdown of MTX2 significantly compromised dynein-mediated retrograde axonal transport in human neurons [118], but whether MTX2 also plays a similar role in the transport of mitochondria in dendrites remains to be investigated.

The mitochondria that are immobilized at the pre- or post-synaptic levels, due to the Ca^2+^-induced loss of motor proteins for the microtubule tracks, can be further recruited into the synaptic compartment by myosin motors that drive the transport of the organelles along actin microfilaments [108]. Thus, depolarization of the plasma membrane was shown to induce actin-based translocation of mitochondria into dendritic spines [109]. Presynaptically, neuronal activity immobilizes axonal mitochondria onto actin microfilaments through Myosin 6 and synthaphilin through a mechanism mediated by AMPK and PAK [18].

A critical role of mitochondria-ER contact sites (MERCS) has also been highlighted in synaptic modulation. Indeed, mitochondria associate closely with the ER, particularly at the dendritic base of spines [28]. MERCs have been shown to regulate ATP production, calcium homeostasis and vesicle release [62], and the dysregulation of mitochondria-ER coupling has been reported in neurodegenerative diseases such as Alzheimer’s [119] and Parkinson’s [120]. MERCs support synaptic activity both in the presynapse and the postsynapse, as well as in astrocytes [121, 122]. The implication of MERCs as mediators of active neurotransmission is connected with changes in calcium and lipid homeostasis, mitochondrial dynamics at the pre- and postsynapse, and regulation of receptors [122]. The intimate contact between mitochondria and ER along dendrites allows for a functional interorganellar coupling and plays a central role in the regulation of the postsynaptic calcium dynamics [123]. Additionally, Miro has been shown to regulate the ER-mitochondria contact sites by clustering along the OMM in association with the mitochondrial contact site and cristae organizing system (MICOS) [124]. This mechanism further connects the maintenance of mitochondrial cristae architecture (regulated by MERCS) and mitochondrial trafficking (by linking TRAK motor adaptors).

### Mitochondrial fission and plasticity of glutamatergic synapses

The alterations in mitochondrial dynamics upon induction of LTP was investigated in cultured hippocampal neurons incubated transiently in a Na^+^-salt solution lacking Mg^2+^ and supplemented with glycine [87]. This protocol of chemical LTP (cLTP) is dependent on the activation of NMDA receptors, enhances spine volume and upregulates the surface expression of AMPA (α-amino-3-hydroxy-5-methyl-4-isoxazole propionic acid) receptors [87]. Under these conditions, there was a rapid upregulation in dendritic mitochondrial fission, accompanied by an increase in mitochondrial matrix Ca^2+^ content. The increase in fission of dendritic mitochondria was triggered by the increase in the [Ca^2+^]_i_, and mediated by activation of Ca^2+^- and calmodulin-dependent protein kinase II (CaMKII) (Fig. 1). Furthermore, cLTP-induced mitochondrial fission was mediated by Drp1 and dynamin 2 [87]. Importantly, inhibition of fission prevented the structural alterations in spines and complementary studies using hippocampal slices also showed a role for mitochondrial fission in LTP of CA1 synapses induced by high-frequency presynaptic stimulation [87]. These results are in accordance with the evidence showing an impairment in spatial working memory in mice with a Drp1 deletion in postmitotic adult mouse forebrain neurons [125]. A key role for mitochondrial fission in synaptic regulation was also shown by the results obtained in cultured hippocampal neurons transfected with a dominant negative form of Drp1 (Drp1-K38A). Expression of this construct downregulated the content in mitochondria, reduced the number of dendritic spines and blocked the activity-induced increase in postsynaptic puncta detected in immunocytochemistry experiments [109]. Furthermore, studies performed in mice with a postnatal deletion of Drp1 showed a decrease in axonal production of ATP, accompanied by an impairment in synaptic transmission and in hippocampus-dependent memory [86]. Interestingly, the knockdown of *Drp1* in D1-medium spiny neurons of the nucleus accumbens blocked cocaine-seeking after self-administration of the drug [88]. PTEN-induced kinase 1 (PINK1) is a key mediator in the activation of Drp1, through the phosphorylation of Ser616, to promote synaptic development and plasticity [126]. In the latter study, the contextual fear memory and spatial memory were found to be impaired in *Drp1*^S616A^ KI mice, showing an important role for this phosphorylation site on Drp1 in learning and memory [126].

Additional studies are required to elucidate the mechanism whereby mitochondrial fission contributes to the LTP of glutamatergic synapses. The structural plasticity of dendritic spines depends on the presence of a stable mitochondrial compartment to fuel local translation required for plasticity events [28]. Therefore, it will be important to investigate to what extent activity-dependent mitochondrial fission events affect the local supply of energy. In addition, whether dendritic mitochondrial fission is related to the local increase in the frequency of mitoflashes, i.e. transient events involving mitochondrial depolarization, production of reactive oxygen species, and matrix alkalinisation [127–132], remains to be investigated. Dendritic mitoflashes in nearby dendritic shafts were proposed to have a signalling role important for the stabilization of structural long-term synaptic potentiation [55].

## BDNF and neuronal mitochondria

Neurotrophins are a family of small, secreted proteins that includes nerve growth factor (NGF), brain-derived neurotrophic factor (BDNF), neurotrophin-3 (NT-3), and neurotrophin-4/5 (NT-4/5). NGF was the first member of this family of proteins to be discovered, in the early 1950s, due to its trophic (survival- and growth-promoting) effects on sensory and sympathetic neurons [133]. However, later studies also showed the presence and function of NGF in the CNS [134]. The discovery of NGF suggested the existence of analogue proteins, which could play similar roles in other regions of the nervous system; this family of proteins was named ‘neurotrophins’ because they have a fundamental role in the survival of their target cells [135]. Neurotrophins signal through their designated tropomyosin-related kinase (Trk) receptors (NGF via TrkA; NT-3 via TrkC; NT4/5 and BDNF via TrkB) as well as through the p75NTR [136].

In mammals, a relatively high expression of BDNF is seen in hippocampal neurons, but also in the frontal cortex, amygdala and hypothalamus [137]. In addition, BDNF is not solely expressed in the brain, being also produced in activated cells of the immune system, endothelial cells, β-cells and muscle tissue [138, 139]. BDNF is transported in blood in high concentrations (ng/ml) in thrombocytes, but the sources and significance of BDNF in the bloodstream have been heavily debated [140]. In fact, although the cells from many different tissues may add to these levels, it is believed that CNS is the main contributor to blood BDNF levels [141]. Evolutionarily, BDNF is a highly conserved protein across species [142] and, presumably, a prerequisite for more complex neuronal structures [135].

Studies performed mainly with cultured neurons have shown that the expression of the BDNF gene is regulated by neuronal activity, and similar alterations were reported in rodents exposed to enriched environments, antidepressant treatment, and exercise [143]. The BDNF gene encodes a precursor peptide, preproBDNF, which is processed by cleavage giving rise to mature BDNF. Following cleavage of the signal peptide, proBDNF is transported to the Golgi for sorting into either the constitutive or regulated secretory vesicles. The proBDNF is converted to BDNF by intracellular and extracellular proteases, and this mature form of the neurotrophin binds to TrkB. The evidence indicating that several aspects of BDNF biology such as transcription, processing, and secretion are regulated by synaptic activity [144] prompted the suggestion that BDNF may regulate activity-dependent forms of synaptic plasticity such as LTP, a sustained enhancement of excitatory synaptic efficacy thought to underlie learning and memory [145].

It was originally proposed that the biological activity of BDNF was limited to the secreted mature form of BDNF, and that proBDNF would serve as an inactive precursor of the neurotrophin. However, accumulating evidence shows that proBDNF is also biologically active, inducing responses that are typically opposite to those triggered by the mature form of the neurotrophin. Thus, proBDNF might induce neuronal apoptosis, via activation of the p75^NTR^ receptor, in contrast with the pro-survival effects of BDNF, mediated by the TrkB receptors [146]. Also, proBDNF induces long-term synaptic depression (LTD) of hippocampal synapses, while BDNF mediates LTP [147, 148].

### BDNF signalling

BDNF/TrkB (Tropomyosin receptor kinase B)-stimulated intracellular signalling is critical for neuronal survival, morphogenesis, and plasticity [143]. Binding of BDNF to TrkB elicits various intracellular signalling pathways, including mitogen-activated protein kinase/extracellular signal-regulated protein kinase (MAPK/ERK), phospholipase C-ɣ (PLC-ɣ), and phosphoinositide 3-kinase (PI3-K)/Akt pathways. Interaction of BDNF with TrkB receptors induces their dimerization followed by transphosphorylation of specific tyrosine residues. The phosphorylation of the receptors allows the interaction with specific adaptor proteins, thereby triggering the downstream intracellular signalling cascades. Activation of PLC-ɣ leads to the formation of inositol 1,4,5-trisphosphate which releases calcium from the endoplasmic reticulum with the consequent stimulation of calcium- and calmodulin-dependent CAMKII. This kinase phosphorylates cAMP response element-binding protein (CREB) thereby activating transcription. In parallel, PLC-ɣ activation leads to the activation of protein kinase C. TrkB receptors are also coupled to the activation of the MAPK/ERK pathway with the downstream activation of transcription through phosphorylation of CREB.

The signalling pathways activated by the BDNF/TrkB complex at the glutamatergic synapses induce changes in dendritic spine morphology. Simultaneous activation of the PI3-K and MAPK/ERK pathways concurrently alter both actin and microtubule dynamics and changes downstream dendrite branching. The role of BDNF in local protein synthesis and in the regulation of RNA metabolism at the synapse, as well as its role in synaptic plasticity and in neuronal development, have been extensively reviewed in [147, 149–151].

### Regulation of mitochondria by BDNF

Neurons continuously change the strength of synaptic communication to encode memories and adapt to experience and to environmental changes. LTP is a key mechanism in this adaptative behaviour. The neurotrophin BDNF is an important mediator of LTP induced by high-frequency presynaptic stimulation, in the hippocampus and in other brain regions [145–147]. The early effects of BDNF in LTP are mediated by posttranslational modification of synaptic components at pre- and postsynaptic level, while the delayed responses require transcription activity and de novo protein synthesis [144–146, 152]. Translation together with regulation of proteasome activity also mediate the facilitatory effects of BDNF-TrkB signalling on CA1 synapses, and protein synthesis is required for consolidation of LTP after infusion of BDNF in the dentate gyrus of anesthetized rats. In addition, BDNF also acts presynaptically to potentiate glutamate release [150, 152–154].

At the presynaptic level, BDNF-induced TrkB signalling stops the transport of mitochondria along the axons and promotes their docking at presynaptic sites by a Ca^2+^-dependent mechanism that also involves the adaptor protein Miro1. The signalling mechanism operating downstream of TrkB receptors to regulate the transport of mitochondria is mediated by an increase in the [Ca^2+^]_i_ through PI3K and PLC-ɣ signalling pathways, and by transient receptor potential canonical (TRPC) channels. More importantly, BDNF-enhanced synaptic transmission is prevented by mutant Miro1 lacking the ability to bind Ca^2+^, indicating an important role for presynaptically accumulated mitochondria on neurotransmission [155].

At the postsynaptic level, BDNF induces the delivery of AMPA and NMDA receptors to the synapse in hippocampal [156] and cortical neurons [157], thereby strengthening excitatory synapses [158]. Both effects depend on protein synthesis [159], and recent studies elucidated a key role for the RNA binding protein hnRNP K in the delivery of transcripts to dendrites which are locally used to synthesize proteins important in synaptic potentiation [152, 158, 160]. BDNF also induces dendritic spine growth through remodelling of spine actin [161]. Furthermore, the increased activity of excitatory synapses induced by BDNF, which raises the [Na^+^]_i_ and [Ca^2+^]_i_, is likely to stimulate plasma membrane transporters (and transporters located in intracellular compartments) to maintain ion gradients. Importantly, all these steps taking place within the postsynaptic compartment are likely to increase the bioenergetic burden on the dendrite, and in particular at the synapse [26]. Therefore, mitochondrial function and dynamics are expected to respond adequately to this burden. For example, a recent study uncovered mitochondria as a key regulator of activity set points in the hippocampus and showed that the inhibition of mitochondrial dihydroorotate dehydrogenase (DHODH) diminishes the mitochondrial maximal respiratory capacity, decreases resting mitochondrial Ca^2+^ and enhances Ca^2+^ buffering during spiking activity in the hippocampus, which in turn decreases the susceptibility to seizures in a model of epilepsy [162]. However, whether BDNF regulates mitochondria dynamics and function at the postsynaptic level has not been properly investigated.

It has been reported that in addition to regulating synaptic plasticity, BDNF can stimulate brain mitochondrial metabolism by increasing the efficiency of respiratory coupling and ATP synthesis [163]. In particular, using a synaptosome preparation (which are isolated nerve endings) and measuring oxygen utilization, BDNF was shown to enhance the respiratory control index (RCI) of rat brain (but not liver) mitochondria, resulting in a 64% increase in the efficiency of respiratory coupling, through the MEK-kinase via complex I [163]. Moreover, the observed effects of BDNF were found not to be due to changes in Ca^2+^ cycling or to a direct action on ATPase activity [163]. A recent study also showed that BDNF induces the local synthesis of the mitochondrial translation initiation factor mtIF3 in the axonal growth cones of cultured hippocampal neurons, followed by the translocation of the protein into mitochondria. The BDNF-induced upregulation in mitochondrial mtIF3 enhances translation into the organelle and mediates BDNF-induced axonal growth [164]. However, the mechanisms by which BDNF regulates mitochondrial function are still unclear. In particular, it remains unknown whether BDNF can regulate mitochondrial transport and distribution in neurons.

More information is available concerning the regulation of mitochondria by NGF in the axonal compartment. The NGF-TrkA signalling is important for the differentiation and maintenance of specific neuronal populations [135, 165]. Their effects are mediated by the MAPK/ERK, phospholipase C-ɣ, and PI3-K/Akt pathways, similarly to what was described above for TrkB signalling. Early studies performed with cultured dorsal root ganglia neurons showed that local stimulation with NGF induces the local accumulation of mitochondria before new axons are formed, by a mechanism dependent on the activity of the PI3-K pathway [166, 167]. In addition, NGF increased locally the mitochondrial membrane potential by a mechanism mediated by the PI3-K and ERK signalling pathways [168]. The two pathways also contribute to the NGF-induced fission of mitochondria along the axons of embryonic chick sensory axons, coupled with collateral branching in vitro [169]. Furthermore, in vivo studies using a dominant negative form of Drp1 showed an impairment in the development of collateral branching of sensory axons in the spinal cord [169]. The ERK pathway was coupled to the regulation of Drp1 GTPase, a fission mediator (see Sect. 2.2), while the PI3-K was important in the control of the effects of actin in mitochondrial fission [169]. Finally, mitochondria fission in the axonal compartment is also an important mediator in the local synthesis of cortactin, an actin-regulatory protein that plays an important role in NGF-induced branching [169]. Whether BDNF plays similar roles in the regulation of axonal mitochondria in CNS neurons remains to be investigated.

## CB-1 cannabinoid receptors and mitochondria

The human cannabinoid receptor 1 (CBR1) and cannabinoid receptor 2 (CBR2) belong to the G protein-coupled receptor (GPCR) family and mediate the biological effects of the natural and endogenous cannabinoid (CB) molecules, as well as the related synthetic analogues [170]. CB1 receptors are highly expressed within the central nervous system, but they are also found at the periphery [171]. On the other hand, CB2 receptors are more abundant in peripheral organs, especially those involved in the immune response [172], although their participation in synaptic plasticity has also been demonstrated [173].

Cannabinoid receptors are coupled to the stimulation of pertussis toxin (PTX)-sensitive G_i/o_ proteins thereby inhibiting adenylyl cyclase (AC) and decreasing intracellular cAMP levels and protein kinase A (PKA) activity. In addition, these receptors activate inwardly rectifying potassium channels (GIRKs) and inhibit N-type and P/Q-type voltage-gated calcium channels, by a mechanism dependent on the activity of G proteins. Given the high expression levels of CB1 receptors on the presynaptic terminal plasma membrane, their activation elicits an hyperpolarization of the plasma membrane which is coupled to the inhibition of neurotransmission, both in excitatory and inhibitory synapses [171, 174, 175]. However, the effects of CB1 receptors are more robust in GABAergic synapses when compared to glutamatergic nerve terminals. In addition, there is compelling evidence showing an intracellular localization of CB1 receptors, complementary to the surface pool of receptors. Thus, CB1 receptors are also found at the endosomal/lysosomal compartments and on the outer mitochondrial membranes (mtCB1) [176–178]. The intracellular CB1 receptors are more abundant in the brain than in peripheral organs, suggesting a key role for mtCB1 in the control of the mitochondrial functions within the nervous system [170].

From the functional point of view, plasma membrane-associated CB1 receptors and the mitochondrial pool of receptors may have different functions as demonstrated in studies performed in the striatonigral circuit. Activation of the former pool of receptors in this circuit is coupled to the inhibition of PKA activity and substance P release, thereby decreasing nociception. On the other hand, the mitochondrial CB1 receptors located at the same nerve endings account for cannabinoid-induced catalepsy, acting through inhibition of intramitochondrial PKA with a consequent reduction in respiration and in substance P release [179]. Future studies should elucidate how the plasma membrane and mitochondrial pools of CB1 receptors are differentially activated by endocannabinoid signalling to modulate pain or motor activity under normal physiological conditions.

A recent study also showed the effect of cannabinoid treatment on the mitochondria architecture in renal proximal tubular cells. This is a fundamental characteristic required for the proper metabolic activity of mitochondria. In vivo administration of CB1 receptor agonists in WT mice impacted the mitochondria morphology inducing mitochondria fragmentation. On the other hand, CB1R^−/−^ mice were protected from these effects, confirming the role of the receptor for this mitochondrial phenotype [180]. The mitochondria fission protein Drp1 is likely involved in the molecular mechanism of this event since the levels of the corresponding mRNA were upregulated and the protein was activated through the decrease in the inhibitory phosphorylation on S637 (PKA-dependent) after the treatment [180]. Additional studies are required to determine whether CB1 receptors induce similar alterations in mitochondrial morphology in neurons.

Activation of the mtCBR1 receptor also induced a significant reduction in the mobility of axonal mitochondria in cultured hippocampal neurons, a critical function for the complex neuronal shape and the high metabolic demand of synapses [181, 182]. Similarly, stimulation of CB1 receptors reduced the fraction of transported mitochondria in enteric nerve fibers [183]. The mechanism underlying the regulation of mitochondrial transport upon activation of cannabinoid receptors has not yet been clarified. mtCBR1 could be in contact with the mitochondrial mobility apparatus and affect its activity upon activation [184]. It is also possible that the alteration of mtCB1-dependent mitochondrial metabolism may affect fundamental characteristics such as the mitochondrial shape and/or the potential of mitochondrial membrane thereby disturbing the proper mobility process. Similarly to the effects in the nervous system, stimulation of the CB1 receptor through 2-arachidonoyl-glycerol (2-AG), anandamide (AEA), and arachidonyl-2-chloroethanolamide (ACEA) (a selective CBR1 agonist) reduces several mitochondrial markers and mitochondrial biogenesis in white adipocytes [185].

### Synaptic regulation by CB-1 receptors

Cannabinoid signalling at synaptic terminals is related to neuronal plasticity events such as LTD and LTP [186]. In the hippocampus, the CB1 receptor is mainly expressed at GABAergic terminals [187] and endocannabinoid signalling has been implicated in the downregulation of GABA release, leading to LTD at inhibitory synapses [188].

CB1 receptors are also found presynaptically on glutamatergic synapses [189] and similarly to the effect on GABAergic terminals, activation of mtCB1 receptors suppress excitatory synaptic transmission in hippocampal CA1 synapses [181]. Electrophysiological studies performed in the CB1 receptor^−/−^ mice model showed no effect of a CB1 receptor agonist in the inhibition of the excitatory synaptic transmission proving the role of the receptor in the regulation of these synapses [190]. Activation of CB1 receptors is also involved in the induction of LTD in hippocampal CA1 synapses as demonstrated pharmacologically: the CB1 receptor selective antagonist AM251 completely blocked this event while the CB1 receptor agonist WIN512212-2 was shown to induce LTD [186]. According to this mechanism, downregulation of the inhibitory currents related to the activation of the CB1 receptors by endocannabinoids facilitate LTP events in neighbouring neurons [191].

In addition to the retrograde signalling, there is growing evidence supporting a postsynaptic mechanism of control of neurotransmission mediated by CB receptors. Activation of the CBR1 at the post-synaptic compartment triggers a cannabinoid-induced hyperpolarization, induced by an increase in the [Ca^2+^]_i_ and activation of the G protein-gated inwardly rectifying potassium (GIRK) channels, lowering the neuronal excitability at the post-synaptic compartments [192].

The effect of CB1 receptors in the regulation of mitochondria was first investigated using CB1^−/−^ mice, which do not allow distinguishing the functional role of neuronal vs glial receptors. Stimulation of CB1 receptors decreased mitochondrial respiration, and consequentially the production of energy in the cells [176, 181], by a mechanism mediated by the inhibition of soluble adenylyl cyclase (sAC). Immunoprecipitation experiments proved the interactions between the CB1 receptors and Gα proteins, and how the latter are released after treatment with cannabinoids. This phenomenon is observed both in neurons and glial cells [175, 181, 193]. The CB1 receptor-induced decrease in the production of cAMP is coupled to downregulation of PKA and consequently alters the functionality of complex I, II and III of the OXPHOS chain [176, 181, 194]. In particular, inhibition of the phosphorylation of the complex I protein NDUFS2 plays a key role in the acute effects of cannabinoids on mitochondrial mobility, synaptic depression and eventually amnesia. The stimulation of intracellular CB1 receptors and inhibition of presynaptic mitochondrial activity, with a consequent reduction in the production of energy by the cells, participate in endocannabinoid-dependent regulation of short-term synaptic plasticity in the hippocampus, providing a possible link between neuronal energy metabolism and specific neuronal functions [176, 181].

**Fig. 3:**
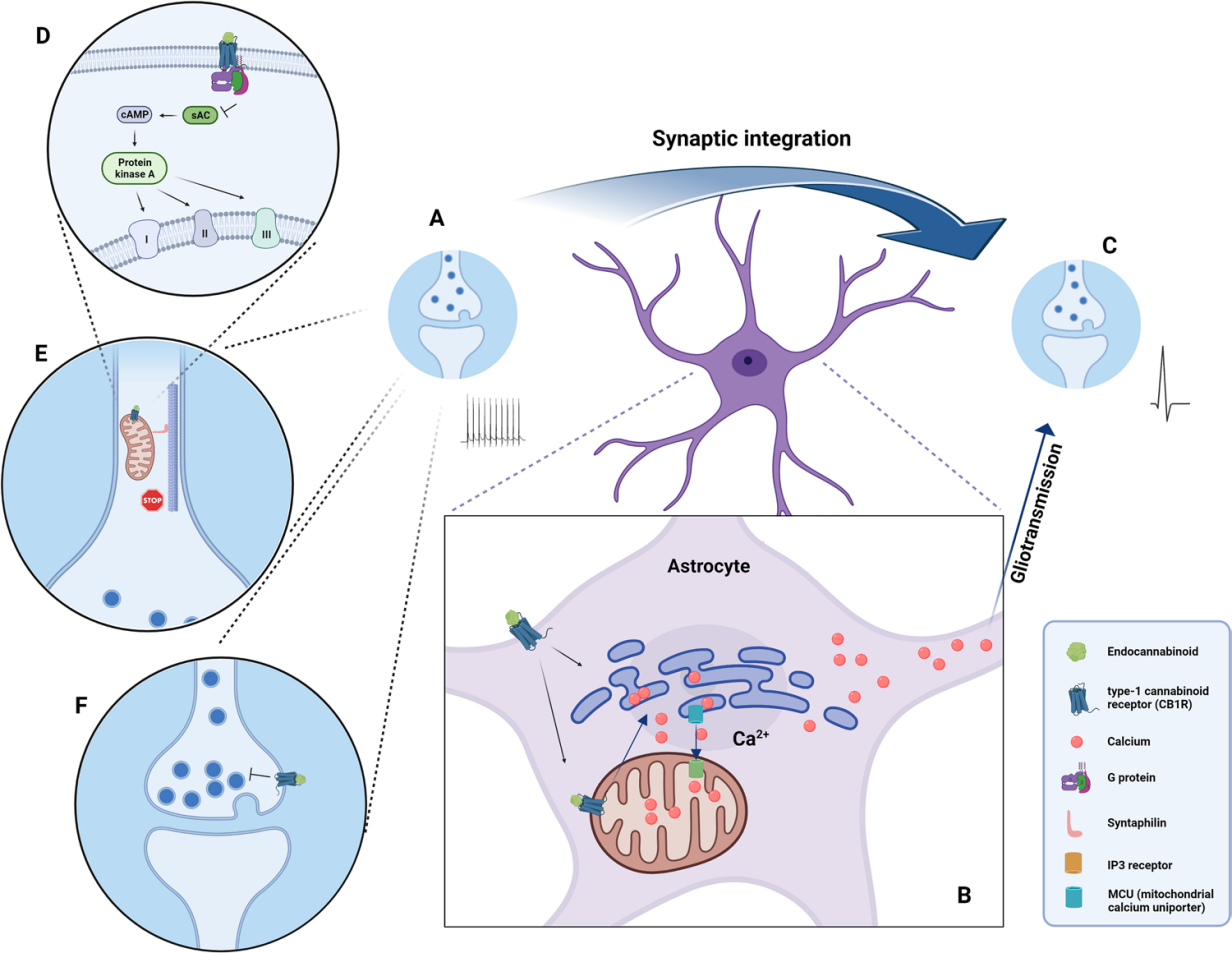
The role of astrocytic surface and mitochondrial CB1 receptors in lateral synaptic potentiation in the hippocampus. Following high-frequency stimulation of a synapse (**A**), the plasma membrane-associated CB1 receptors in a contacting astrocyte elicit Ca^2+^ release from the endoplasmic reticulum (**B**); concomitant stimulation of mitochondrial CB1R (mtCB1R) allows the entry of Ca^2+^ into the mitochondria (**B**), via the mitochondrial calcium uniporter and using MERCS. It was proposed that the uptake of Ca^2+^ into the mitochondria determines the dynamics of the [Ca^2+^]_i_ changes in astrocytes, to induce lateral synaptic potentiation (**C**). The activation of the mtCB1R, which is located in the outer mitochondrial membrane, leads to the inhibition of the sAC (soluble adenylyl cyclase), in turn resulting in a decrease of mitochondrial respiration (**D**) and consequently in a decrease in energy production. Moreover, the synaptic transmission leads to a stop in mitochondrial trafficking/recruiting in the pre-synaptic compartment (**E**). The membrane CB1R in the pre-synaptic neuron additionally signals an inhibition in neurotransmitter release (**F**). (Created with BioRender.com)

More recent studies also showed a pivotal role of astrocytic surface and mitochondrial CB1 receptors in lateral synaptic potentiation in the hippocampus, via the determinant role of astroglial MERCS in synaptic integration [121]. The plasma membrane-associated CB1 receptors in astrocytes are mainly coupled to G_αq/o_ proteins that activate phospholipase C, with the downstream production of inositol 1,4,5-trisphosphate which releases Ca^2+^ from the endoplasmic reticulum, and stimulation of protein kinase C [195]. Concomitant stimulation of mitochondrial CB1 receptors allows the entry of Ca^2+^ into the mitochondria through the mitochondrial MCU, through a mechanism mediated by Akt-dependent phosphorylation of mitochondrial Ca^2+^ uptake protein (MICU1), an important modulator of the MCU. It was proposed that the uptake of Ca^2+^ into the mitochondria determines the dynamics of the [Ca^2+^]_i_ changes in astrocytes, to induce lateral synaptic potentiation [121] (see Fig. 3). In addition, the reduction of glucose metabolism and the increased production of lactate in the brain induced by activation of astrocytic mitochondrial CB1R impairs the behavioural responses in social interaction assays [193].

In particular, activation of astroglial mitochondrial CB1 receptors decreases the phosphorylation of the NDUFS4 subunit of the mitochondrial complex I on Ser173, with consequent destabilization of the N-module that contains a binding pocket for O_2_ molecules that accept electrons from NADH(H^+^) to generate mitochondrial reactive oxygen species. Therefore, stimulation of mitochondrial CB1 receptors decreases the stability and activity of complex I, with a consequent reduction in the production of reactive oxygen species by astrocytes, and affects the production of lactate through glycolysis by a mechanism involving the hypoxia-inducible factor-1α (HIF-1α) transcriptional pathway. Ultimately, stimulation of mtCB1 receptors results in neuronal redox stress and impairment of the behavioural social responses [193].

The administration of delta-9-tetrahydrocannabinol (THC), a cannabinoid receptor agonist, after training in the object recognition and the context-recognition tasks was demonstrated to induce amnesia [196]. Since mitochondrial-ATP production is crucial for the proper neuronal network transmission [197], the impairment in mitochondrial energy production chain following the administration of cannabinoids may account for the observed learning deficits and memory impairment. This hypothesis is supported by the results showing that deletion of the mtCB1 receptor prevents cannabinoid-induced decrease in memory formation, as determined using the novel object recognition test [181]. More recent studies showed that the reduced CB1 signalling in CB1-KO mice leads to a decrease in mitophagy and abnormal mitochondrial morphology in hippocampal neurons during aging [198].

Together, the data collected so far confirms a pivotal role for cannabinoids and their receptors in the modulation of mitochondrial functions. Although there appears to be a clear link between changes in cannabinoid-induced mitochondrial activity and synaptic alterations, the specific mechanisms involved remain to be determined. A deeper knowledge of the relationship between cannabinoid signalling and mitochondria remains a challenge.

## IGF-1 receptor and mitochondria

Insulin-like growth factor 1 receptors (IGF1-R) are widely expressed in the central nervous system, playing important roles in the control of brain metabolism and energy homeostasis, in brain development, injury repair, and in cognition and mood regulation (reviewed in [199, 200]). These receptors bind to IGF-1 and IGF-2 with a high affinity and show a lower affinity for insulin. Binding of IGF-1 to the α-subunit of IGF-1R leads to a conformational change in the β-subunit with a concomitant activation of the tyrosine kinase activity of the receptors [201]. The signalling mechanisms activated by IGF-1R are similar to those described above for TrkB receptors and include the activation of the Ras-ERK pathway and the PI3-K pathway [202].

Studies performed in cultured hippocampal neurons and in CA1 synapses in hippocampal slices showed that the activity of IGF-1R suppresses spontaneous excitatory neurotransmission under resting conditions, but upregulates the evoked glutamatergic synaptic transmission [203]. The differential effects of IGF-1R under resting conditions and upon synaptic stimulation correlated with a distinct regulation of the mitochondrial Ca^2+^ concentration ([Ca^2+^]_mito_) and in the presynaptic levels of ATP. Thus, inhibition of IGF-1R under resting conditions decreased [Ca^2+^]_mito_ but did not affect the presynaptic levels of ATP. Therefore, the IGF-1R tone constrains the resting cytosolic free [Ca^2+^]_i_ and the spontaneous release of neurotransmitters through buffering of the mitochondrial Ca^2+^. In contrast, downregulation of IGF-1R under conditions of synaptic activity decreased presynaptic ATP without changing [Ca^2+^]_mito_, maintaining action potential-evoked presynaptic [Ca^2+^]_i_ transients [203]. Together, these results show that a tonic activity of IGF-1R maintains the evoked-to-spontaneous ratio of glutamatergic neurotransmission in hippocampal synapses. This effect is mediated by the regulation of mitochondria, but the underlying molecular mechanism remains to be investigated.

Moreover, recently, exogenous IGF-1 was found to alleviate mitochondrial dysfunction and inhibition of CREB/PGC-1α signalling in the hippocampus of high-fat diet-fed mice [204]. In the same study, the authors showed that IGF-1 alleviated depression-like behaviours in these animals. However, in this case the animals were treated with IGF-1 for four weeks and, therefore, the mechanisms of synaptic and mitochondrial regulation may be distinct from those observed under short-term treatment.

## Closing remarks and future perspectives

Mitochondrial dynamics is tightly regulated and has a significant impact on mitochondrial function. This is noticeable from the result of primary defects in mitochondrial fusion and fission which are involved in some diseases of the nervous system, such as Charcot-Marie-Tooth disease type 2A, dominant optic atrophy and some forms of intractable epilepsy [205]. The distinct size and dynamics of dendritic and axonal mitochondria also show a differential regulation of the organelle in the two compartments that largely remain to be investigated. In particular, the understanding of the mechanisms that control the local movement of mitochondria at the synapse will require a detailed elucidation of the dynamics of Ca^2+^ ions and metabolites under the same conditions and concerning the interactions of mitochondria with adaptor proteins and molecular motor that travel along actin and microtubules. Future studies should also elucidate the role of surface receptors in the coordinated regulation of ATP production by mitochondria and glycolytic enzymes at the synapse. The cross-talk between different surface receptors involved in the regulation of mitochondria may add an additional layer of complexity to the local regulation of the synaptic population of the organelles.

In addition to the heterogeneity between subcellular neuronal compartments, there is also a distinct energy utilization and mitochondrial activity among brain regions [206], suggesting that neuronal mitochondria may undergo differential regulation depending on the type of neurons. This is a question that requires further investigation, together with a better understanding of the protein composition of mitochondria in different brain regions.

The full elucidation of the mechanisms involved in the regulation of synaptic mitochondria by neuronal activity and by surface receptors, and the downstream functional consequences, will allow elucidating of how this organelle contributes to the maintenance of functional neurons and the brain under normal physiological conditions. Importantly, the regulation of neuronal synaptic mitochondria may depend not only on the pattern of neuronal activity by also on neuron-glia interactions. Elucidation of these regulatory mechanisms will require the in vivo analysis of mitochondrial dynamics, in contrast with most studies in the field performed up to now, in which the organization of mitochondria has been studied mainly in cultured neurons and in brain slices.

## Data Availability

Not applicable.
